# Supplementary material for: Orchestrated ensemble activities constitute a hippocampal memory engram
Source: Nat Commun. 2019 Jun 14;10:2637. doi: 10.1038/s41467-019-10683-2 (PMC6570652; doi:10.1038/s41467-019-10683-2)
Supplement: Supplementary file 1 — Supplementary Information [file 41467_2019_10683_MOESM1_ESM.pdf]

Supplementary information

Orchestrated ensemble activities constitute a hippocampal memory engram

Ghandour et al. 2019

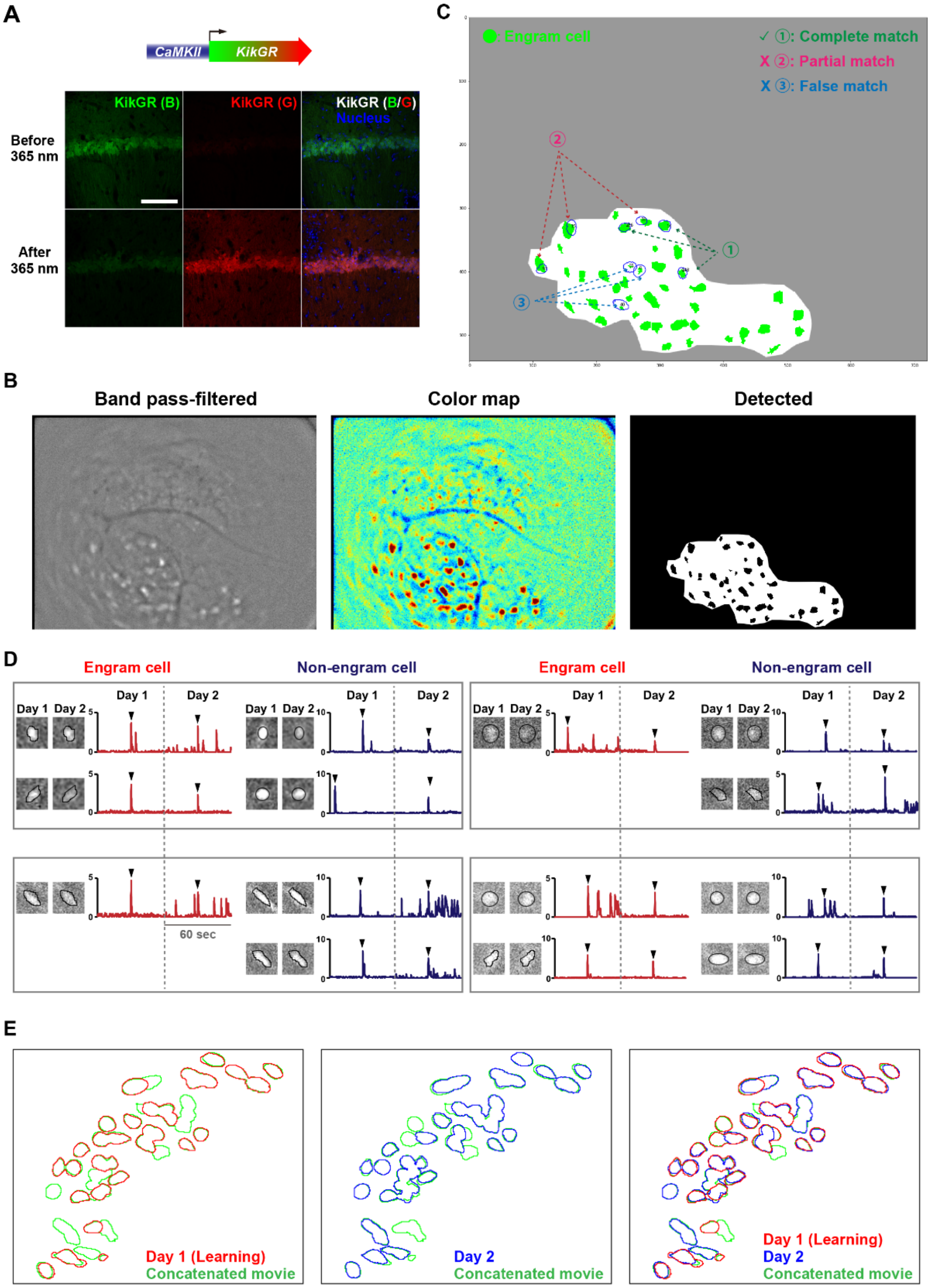

Supplementary Figure 1

Supplementary Figure 1. Validation of the process for identifying and extracting calcium transients from engram cells and non-engram cells.

(A) Representative image of KikGR expressed under the control of the CaMKII promoter in CA1 of WT mice before (upper) and after (lower) photoconversion by 365 nm light exposure in vitro (scale bar, 100 μm).

(B) Representative snapshot image of $KikGR^+$ expression after applying a band-pass filter (see Methods) (left), heat map (center), and automatic identification of $KikGR^+$ (right) (see Methods).

(C) Criteria for selecting engram cells for further analysis. Only cells showing a complete match between the $KikGR^+$ cell from the snapshot (green filled contours) and the automatic calcium detection system (blue contours) (category 1) (i.e., cells double positive for c-fos tet tagging and G-CaMP7 fluorescence) were considered for further analysis. Partially matching cells (category 2) and false matches (category 3) (i.e., detected fluorescence from the snapshot (green filled contours) that did not match cell morphology) were excluded from further analysis.

(D) Representative ROIs for some engram and non-engram cells showing their spatial location and traces over 2 days of recording indicated in red (engram) and blue (non-engram). These examples were extracted from four mice at random. Arrowheads indicate the timing of the Ca^{2+} events used to evaluate the cell location.

(E) Spatial footprints of temporally matched cells detected by the HOTARU system from the concatenated movie and from single videos were overlaid to assess spatial shift and cell morphology stability across sessions. Cells detected from the concatenated movie are shown in green (left, center, right), those from the day 1 movie are shown in red (left, right), and those from the day 2 movie are shown in blue (center, right).

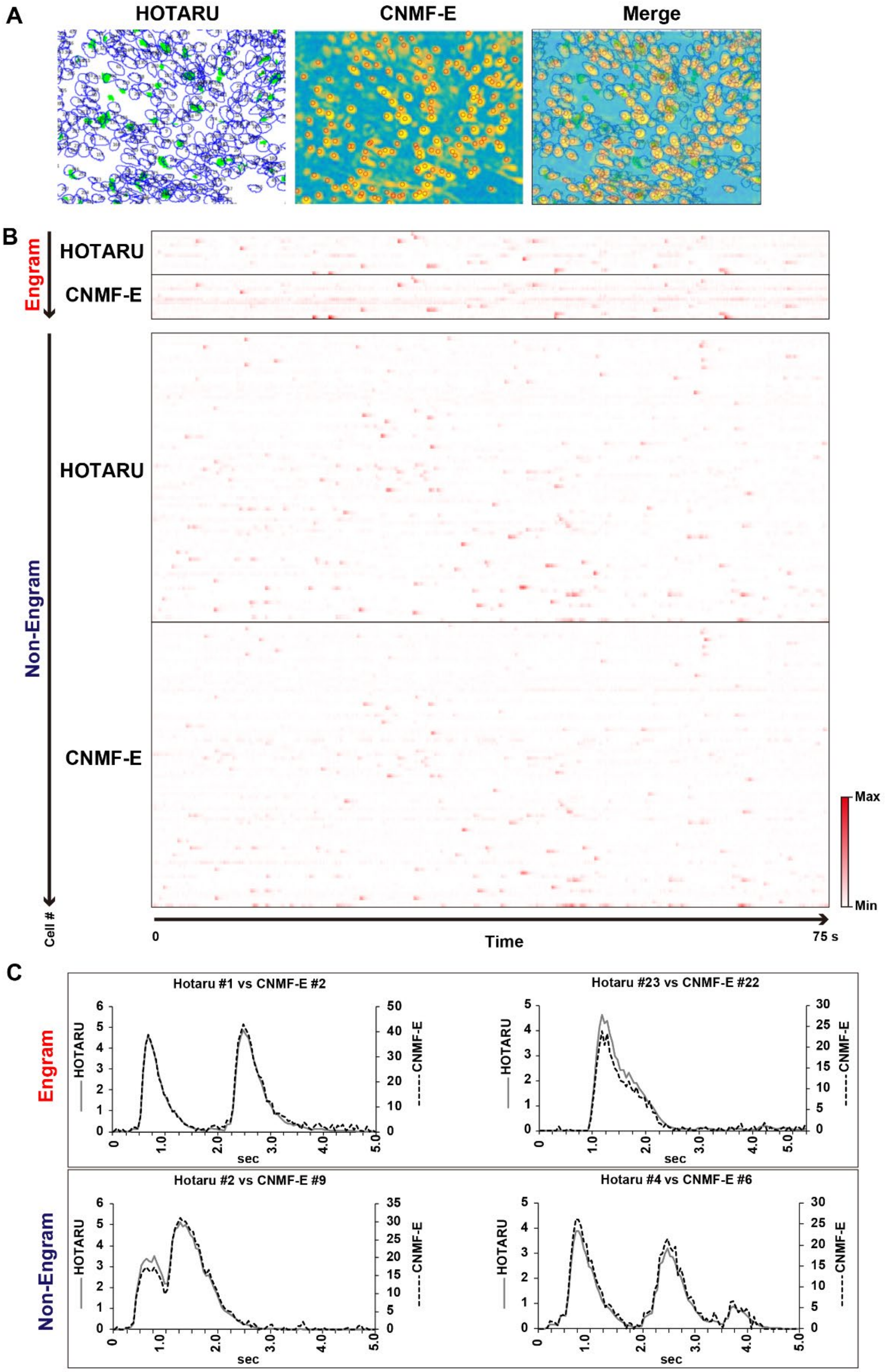

Supplementary Figure 2

Supplementary Figure 2. Automatic sorting system output was comparable to that from the CNMF-E analysis.

(A) Representative image of all cells detected from a single animal by an automatic calcium activity detection system, HOTARU (left) and the CNMF-E system (center); both images overlaid (right).

(B) Calcium activity traces in all engram (top) and non-engram cells (bottom) as detected by both systems.

(C) Representative calcium traces for engram (top) and non-engram (bottom) cells, as detected by both systems.

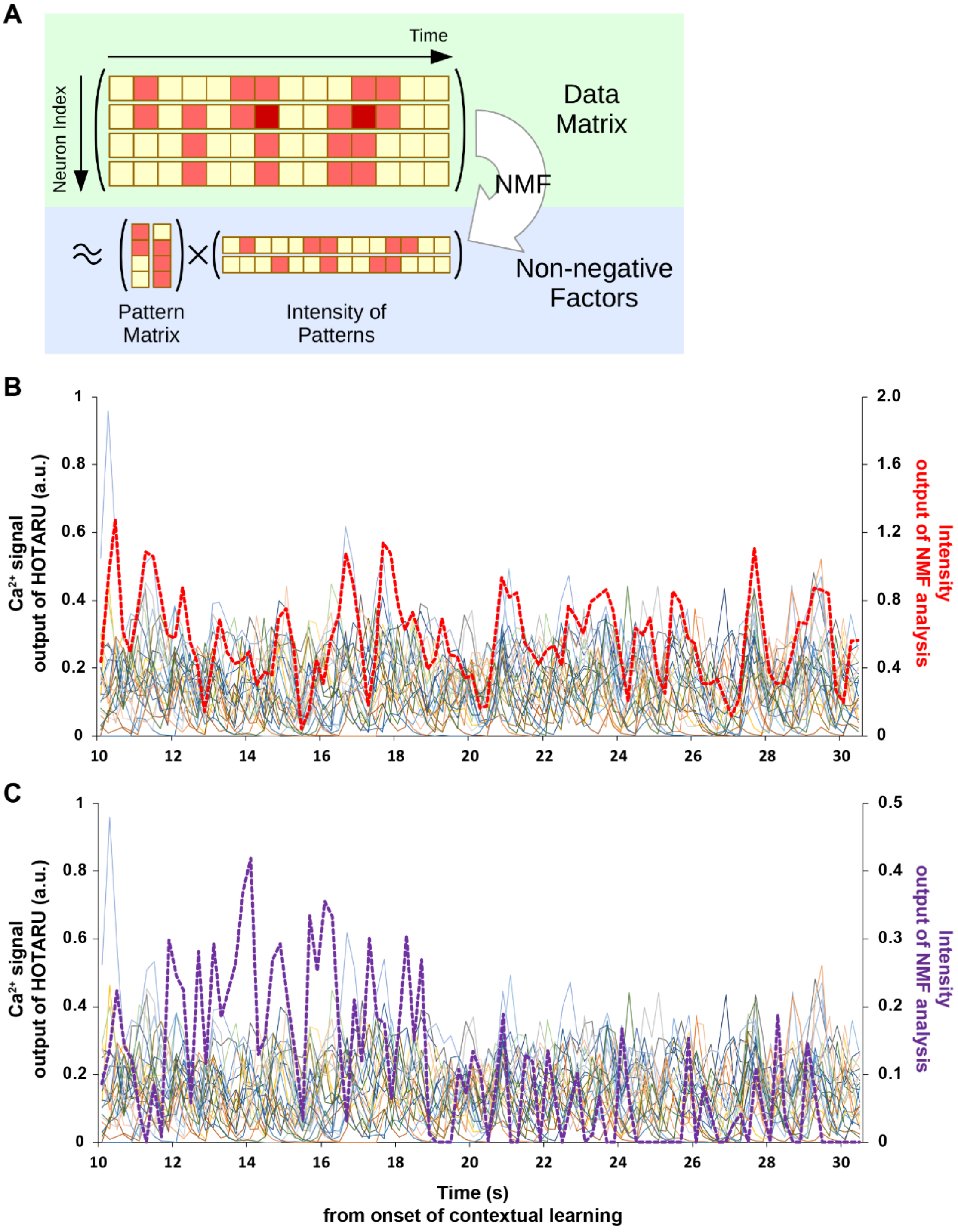

Supplementary Figure 3

Supplementary Figure 3. The intensity of a particular pattern specifically matches the activity of the pattern neurons.

(A) Schematic diagram showing non-negative matrix factorization (NMF) analysis.

(B) Pattern X intensity (secondary *y*-axis) and the calcium transients of pattern X-participating cells (primary *y*-axis) across time (*x*-axis).

(C) Relationship between cells in pattern X and the intensity of pattern Y.

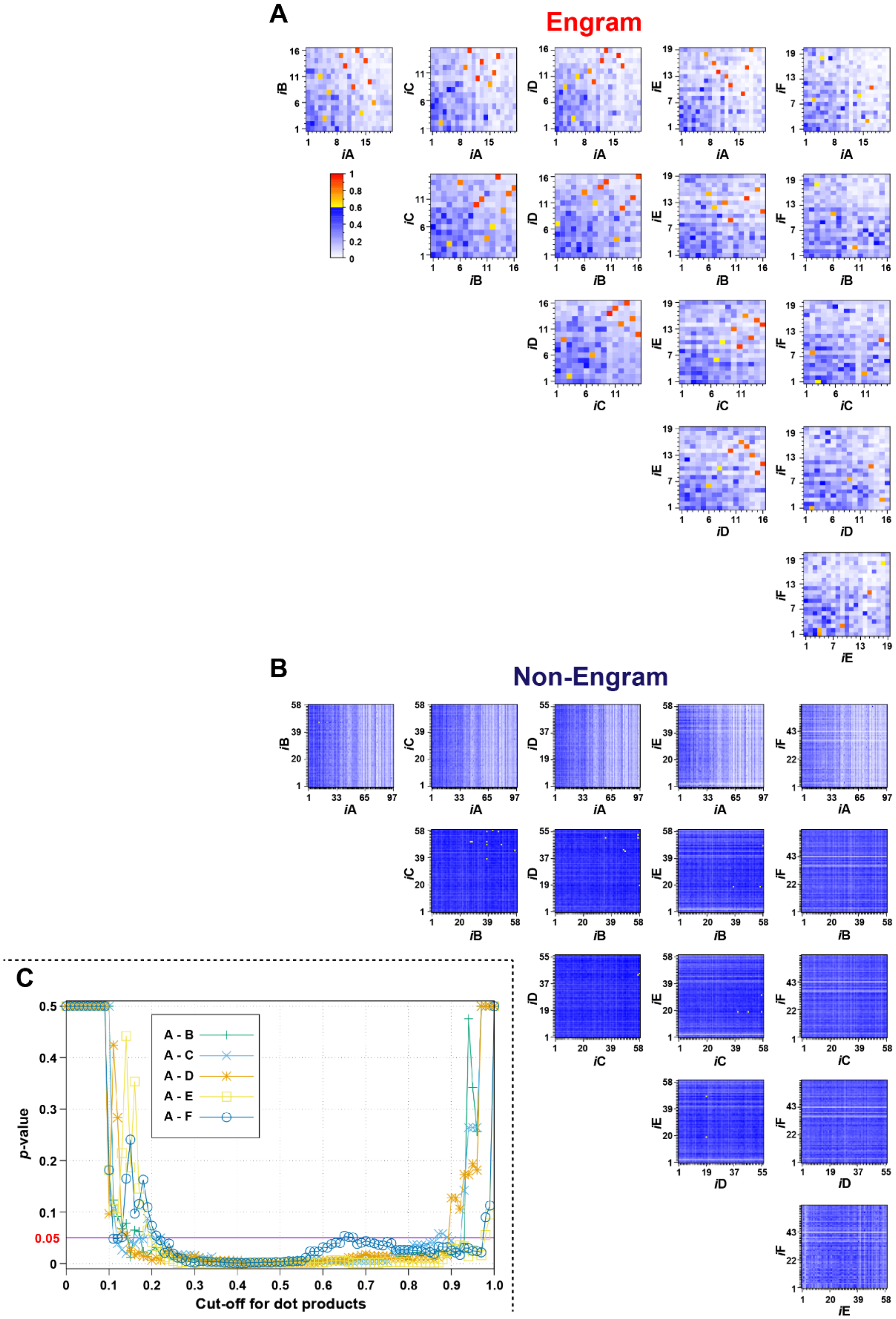

Supplementary Figure 4

Supplementary Figure 4. Matching patterns (dot product > 0.6) across sessions in both engram and non-engram cells.

(A and B) Representative results of the dot product analysis of NMF-extracted patterns for both engram (A) and non-engram (B) cells.

(C) Summary of *p*-values from paired t test, one-tailed, for different dot product thresholds, showing the *p*-value for the difference between the MSs for engram and non-engram cells in each session (at several dot product thresholds).

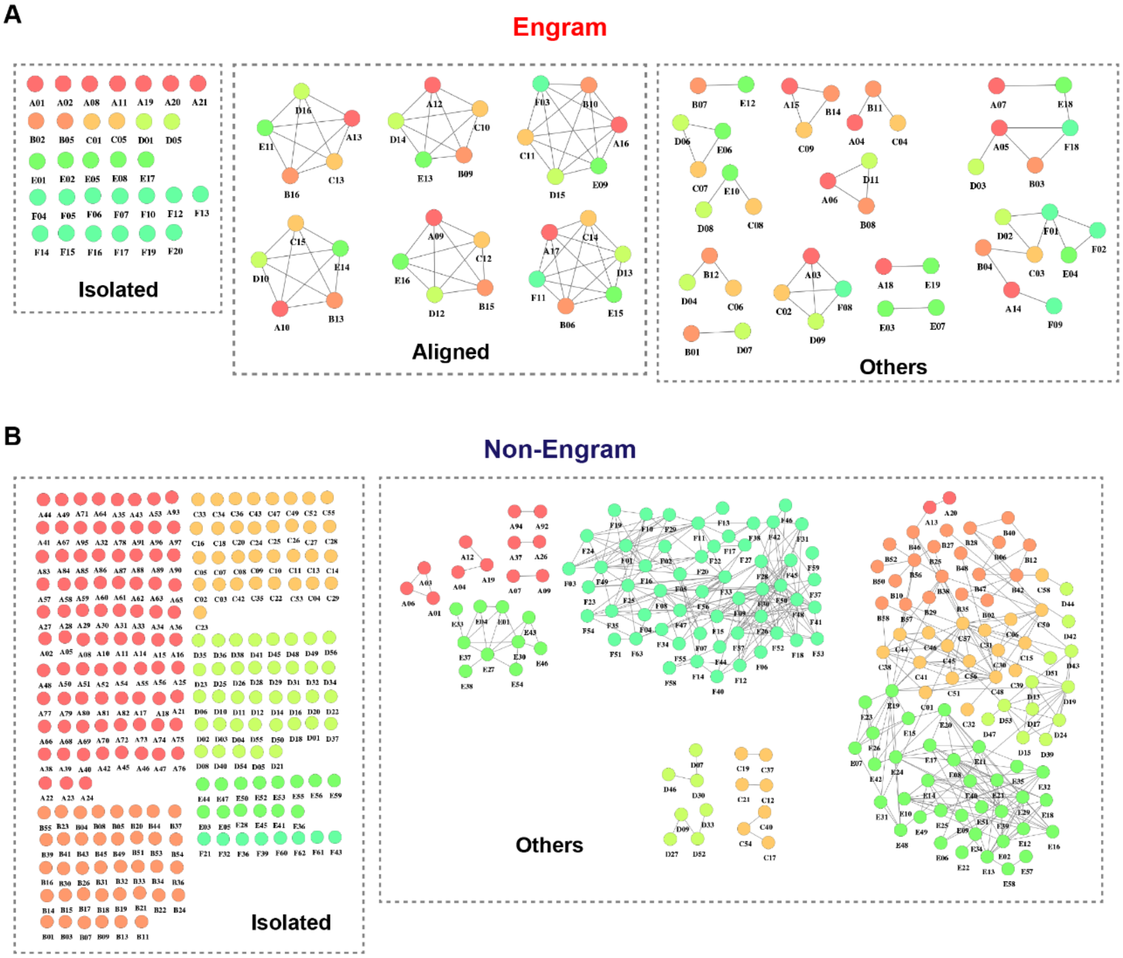

Supplementary Figure 5

Supplementary Figure 5. Engram pattern pairs are more similar across sessions.

(A and B) Schematic diagrams showing pattern networks for (A) engram cells and (B) non-engram cells. Depending on the dot product values across sessions, several categories of pattern can form: isolated (no pattern match in other sessions), aligned (patterns match across sessions), and other.

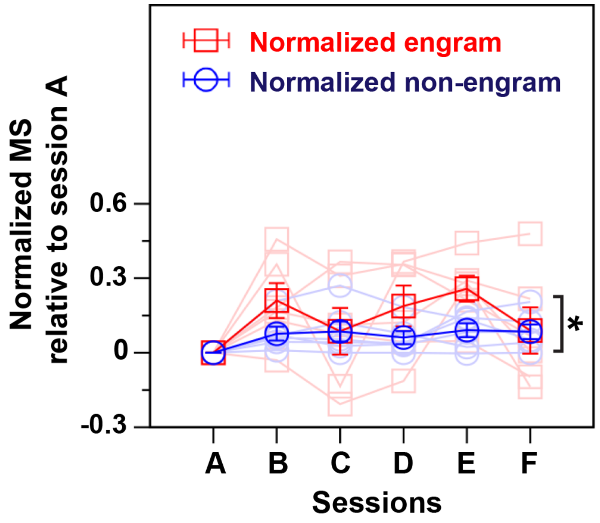

Supplementary Figure 6

Supplementary Figure 6. Differences between normalized engram and non-engram across sessions.

MS across sessions (with respect to session A) for normalized engram and non-engram data. $n = 6$; two-way repeated-measures ANOVA, $F_{(1, 30)} = 5.949$, $p = 0.0209$ (engram vs non-engram). $*p < 0.05$. Data represent the mean ± s.e.m.

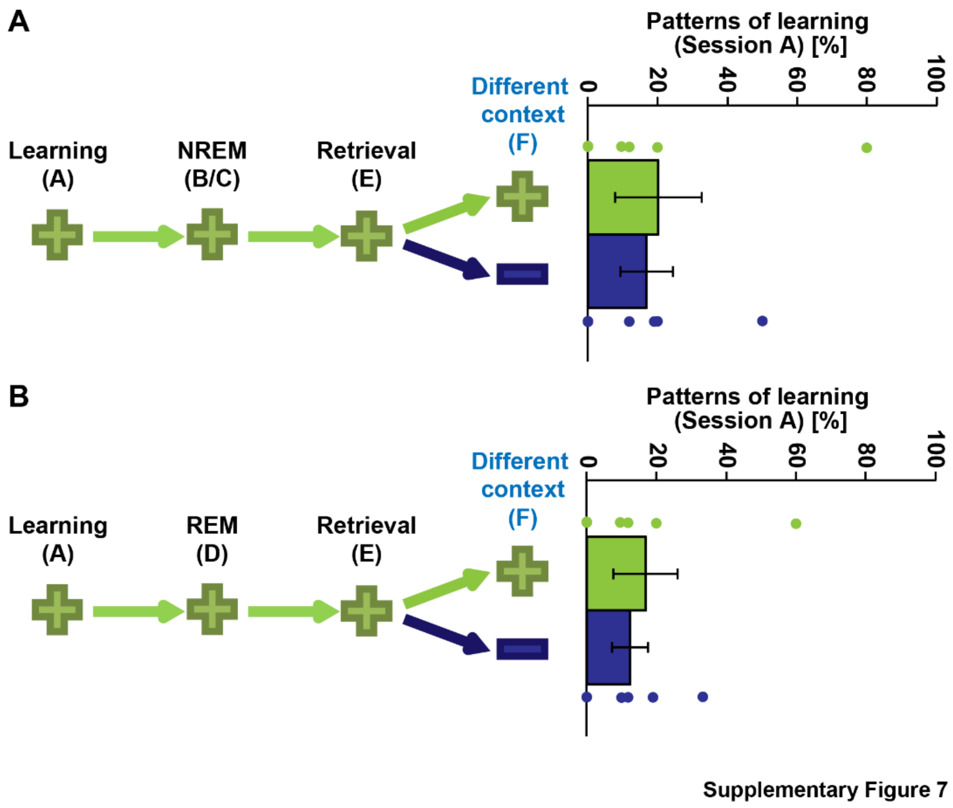

Supplementary Figure 7. Engram sub-ensembles specific to context A.

(A and B) Similar analysis to that used in Fig. 6a and b in the main paper. Engram sub-ensembles reactivated during NREM (A) or REM (B) sleep and retrieval were analyzed. A paired *t*-test, two-tailed, showed no statistically significant differences. $n = 6$. Data represent the mean ± s.e.m.

Animal ID	Engram cells	Non-engram cells	Total	Data are used for
NV1	48	353	401	Fig. 3d
NV2	29	474	503	Fig. 3d
NV3	44	405	449	Fig. 3d
NV4	46	418	464	Fig. 3a–d, S2
NV5	19	159	178	Fig. 3d, 4c–d, 5d–i, 6a–b, S6, S7
NV6	30	319	349	Fig. 3d, 4c–d, 5d–i, 6a–b, S1D, S6, S7
NV7	12	518	530	Fig. 3d, 4b–d, 5d–i, 6a–b, S1D, S6, S7
NV8	66	947	1013	Fig. 3d, 4c–d, 5b–i, 6a–b, S1D, S3B–C, S4, S5, S6, S7
NV9	41	492	533	Fig. 2d–e, 4c–d, 3d, 5d–i, 6a–b, S6, S7
NV10	16	191	207	Fig. 3d, 4c–d, 5d–i, 6a–b, S1B–D, S6, S7
Average	35.1	427.6	462.7	
Percentage	8.09% (±0.84)	91.91	100	

Supplementary Table 1: Number of engram and non-engram cells detected from animals used in this study.
