# Supplementary material for: Orchestrated ensemble activities constitute a hippocampal memory engram
Source: Nat Commun. 2019 Jun 14;10:2637. doi: 10.1038/s41467-019-10683-2 (PMC6570652; doi:10.1038/s41467-019-10683-2)
Supplement: Supplementary file 3 — Reporting Summary [file 41467_2019_10683_MOESM3_ESM.pdf]

nature research

Corresponding author(s): Kaoru Inokuchi

Reporting Summary

Nature Research wishes to improve the reproducibility of the work that we publish. This form provides structure for consistency and transparency in reporting. For further information on Nature Research policies, see Authors & Referees and the Editorial Policy Checklist.

Statistical parameters

When statistical analyses are reported, confirm that the following items are present in the relevant location (e.g. figure legend, table legend, main text, or Methods section).

n/a	Confirmed	
☐	☒	The exact sample size (*n*) for each experimental group/condition, given as a discrete number and unit of measurement
☐	☒	An indication of whether measurements were taken from distinct samples or whether the same sample was measured repeatedly
☐	☒	The statistical test(s) used AND whether they are one- or two-sided<br>*Only common tests should be described solely by name; describe more complex techniques in the Methods section.*
☐	☒	A description of all covariates tested
☐	☒	A description of any assumptions or corrections, such as tests of normality and adjustment for multiple comparisons
☐	☒	A full description of the statistics including central tendency (e.g. means) or other basic estimates (e.g. regression coefficient) AND variation (e.g. standard deviation) or associated estimates of uncertainty (e.g. confidence intervals)
☐	☒	For null hypothesis testing, the test statistic (e.g. *F*, *t*, *r*) with confidence intervals, effect sizes, degrees of freedom and *P* value noted<br>*Give P values as exact values whenever suitable.*
☒	☐	For Bayesian analysis, information on the choice of priors and Markov chain Monte Carlo settings
☒	☐	For hierarchical and complex designs, identification of the appropriate level for tests and full reporting of outcomes
☒	☐	Estimates of effect sizes (e.g. Cohen's *d*, Pearson's *r*), indicating how they were calculated
☐	☒	Clearly defined error bars<br>*State explicitly what error bars represent (e.g. SD, SE, CI)*

Our web collection on statistics for biologists may be useful.

Software and code

Policy information about availability of computer code

Data collection	Please see "Calcium imaging data acquisition, processing, and cell sorting" section in Methods. Calcium imaging data were collected using nVista acquisition software (Inscopix, CA) , and calcium transients were extracted by a fully automated calcium sorting algorithm, HOTARU system, which is referenced.
Data analysis	NMF analysis (Fig. 5) were performed using a custom made program that can be found in https://github.com/fccaa/NMF_custom.

For manuscripts utilizing custom algorithms or software that are central to the research but not yet described in published literature, software must be made available to editors/reviewers upon request. We strongly encourage code deposition in a community repository (e.g. GitHub). See the Nature Research guidelines for submitting code & software for further information.

Data

Policy information about availability of data

All manuscripts must include a data availability statement. This statement should provide the following information, where applicable:

- Accession codes, unique identifiers, or web links for publicly available datasets
- A list of figures that have associated raw data
- A description of any restrictions on data availability

The data that support the findings of this study are available from the corresponding author upon request.

Field-specific reporting

Please select the best fit for your research. If you are not sure, read the appropriate sections before making your selection.

☒ Life sciences ☐ Behavioural & social sciences ☐ Ecological, evolutionary & environmental sciences

For a reference copy of the document with all sections, see nature.com/authors/policies/ReportingSummary-flat.pdf

Life sciences study design

All studies must disclose on these points even when the disclosure is negative.

Sample size	Sample size was determined based on relevant previous literatures that perform calcium imaging.
Data exclusions	All analyzed data are included in the current study.
Replication	The conclusions we made are based on the analysis of data from each mouse and the reproducibility of the results across all the mice incorporated in the study.
Randomization	This is not relevant in the current study, as all mice contain both engram and non-engram cells.
Blinding	Blinding is not relevant in the current study.

Reporting for specific materials, systems and methods

Materials & experimental systems

n/a	Involved in the study
☐	☒ Unique biological materials
☐	☒ Antibodies
☒	☐ Eukaryotic cell lines
☒	☐ Palaeontology
☐	☒ Animals and other organisms
☒	☐ Human research participants

Methods

n/a	Involved in the study
☒	☐ ChIP-seq
☒	☐ Flow cytometry
☒	☐ MRI-based neuroimaging

Unique biological materials

Policy information about availability of materials

Obtaining unique materials	The lentiviral vectors used in this study are available from the corresponding author upon request.

Antibodies

Antibodies used	Please see "Immunohistochemistry section" in Methods. Rabbit anti-GFP (1:1000, Molecular Probes) and donkey anti-rabbit IgG-AlexaFluor 488 secondary antibody (Molecular Probes, A21206 and A11056).
Validation	Antibodies used were previously used in a study from the same lab (Ohkawa et al., Cell reports, 2015).

Animals and other organisms

Policy information about studies involving animals; ARRIVE guidelines recommended for reporting animal research

Laboratory animals	c-fos-tTA mice and c-fos-tTA x Thy1-G-CaMP7 mice, C57BL/6J background, Male, 12-20 weeks.
Wild animals	The study did not involve wild animals.
Field-collected samples	The study did not involve work samples collected from the field.
